# Comparison of patient, hospitalization and center characteristics from the EMMY centers, IFM centers, and all multiple myeloma centers in France

**DOI:** 10.46989/001c.161361

**Published:** 2026-05-26

**Authors:** Decaux Olivier, Ronan GARLANTEZEC, Aurore PERROT, Margaret MACRO, Bruno ROYER, Karim BELHADJ MERZOUG, Laure VINCENT, Thomas CHALOPIN, Laurent FRENZEL, Arthur BOBIN, Mohamad MOHTY, Cyrille TOUZEAU, Patricia ZUNIC, Ronan LE CALLOCH, Florence LACHENAL, Murielle ROUSSEL, Valentine RICHEZ-OLIVIER, Joel FLEURY, Elena LOPPINET, Isabelle LEDUC, Mourad TIAB, Jacques DELAUNAY, Jean GUTNECHT, Selim CORM, Fabienne BAZIN, Pippa McKelvie-Sebileau, Sandrine ROLLET, Heloise MOUTON, Laurent GIBEL, Chanaz LOUNI, Ariane BOUMENDILE, Cyrille HULIN

**Affiliations:** 1 Service d’hématologie clinique, CHU de Rennes, Rennes, France; 2 MOBIDIC - Microenvironment and B-cells: Immunopathology, Cell Differentiation, and Cancer, CHU Rennes, Rennes, France; 3 Santé publique et épidémiologie, CHU de Rennes, Rennes, France; 4 Service d’hématologie, Institut Universitaire du Cancer de Toulouse-Oncopole, Toulous, France; 5 Service d’hématologie, CHU Caen, Caen, France; 6 Service d’Immuno-hématologie, Hôpital Saint Louis, Paris, France; 7 Unité Fonctionnelle Hémopathies Lymphoïdes CHU. Henri Mondor, Créteil, France; 8 Department of Clinical Hematology, Montpellier University Hospital Center, Montpellier, France; 9 Service d’hématologie et Thérapie Cellulaire, Centre Hospitalier Universitaire (CHU) Tours, Hôpital Bretonneau, Tours, France; 10 Service d’hématologie adulte - Responsable centre régional de traitement de l’hémophilie CRTH - Centre national de référence des mastocytoses CEREMAST, Hôpital Necker-Enfant Malade, Paris, France; 11 Department of Hematology, University of Poitiers, Poitiers, France; 12 Department of Haematology, Saint Antoine Hospital, Paris, France; 13 Hematology Department, University Hospital Hôtel-Dieu, Nantes, France; 14 Service d’hématologie, Hôpital Saint-Pierre, France; 15 Hematology, CH de Cornouaille, Quimper Concarneau, Concarneau, France; 16 Department of Haematology, CH Pierre Oudot, Bourgoin-Jallieu, France; 17 Department of Hematology, Hôpital Universitaire de Limoges, Limoges, France; 18 Department of Hematology, Hôpital Universitaire de Nice, Nice, France; 19 Hematology Department, Clermont-Ferrand Cancer Institute, France; 20 Service d’hématologie, Hôpital Robert Schuman, Metz, France; 21 Hematology Department, CH d’Abbeville, Abbeville, France; 22 Centre Hospitalier Départemental Vendée, La Roche sur Yon, France; 23 Department of Onco-hematology, Hôpital Privé Le Confluent, Nantes, France; 24 Department of internal medicine, CHI Frejus Saint Raphaël, Frejus, France.; 25 Medipole de Savoie, Challes les Eaux, France; 26 Horiana Health Data Consulting, Bordeaux, France; 27 Intergroupe Francophone du Myélome, Paris, France; 28 Hématologie, Hôpital Haut-Lévêque, Pessac, France

**Keywords:** multiple myeloma, registry cohort study, longitudinal cohort, real-world evidence

## Abstract

**Background:**

Multiple myeloma (MM) is an incurable hematological malignancy with increasing prevalence. While randomized controlled trials have demonstrated survival improvements, they underrepresent older and comorbid patients. Real-world data from observational cohorts are therefore essential.

**Objective:**

To assess the comparability of patient, hospitalization and center characteristics for patients treated in centers included in the French Epidemiology of Multiple MYeloma (EmmY) cohort with those treated in centers of the French-speaking Intergroupe Francophone du Myélome (IFM) network (including EmmY) and those treated in all centers treating MM in France between 2017–2023.

**Methods:**

We analyzed French national hospital discharge data (PMSI) to identify adults hospitalized with MM (ICD-10 C90) between August 2017 and December 2023. Patient demographics, comorbidities, hospitalization characteristics, and center profiles were compared between the three cohorts using standardized differences.

**Results:**

We identified 69,276 patients (including 50,243 IFM and 33,785 EmmY), 1,871,369 hospitalizations (including 1,484,288 IFM and 1,022,462 EmmY), and 323 centers (including 118 IFM and 70 EmmY). EmmY centers were larger and treated a higher mean proportion of MM patients (2.7%) per center than IFM (2.4%) and all MM centers (1.3%). No clinically meaningful differences were observed between EmmY and IFM centers regarding patient age, sex, comorbidities, treatment patterns, or hospitalization outcomes.

**Conclusions:**

The EmmY cohort is highly comparable to patients treated in specialized MM centers and those within the broader French MM population, supporting its validity as a robust real-world data source. It is well-suited for evaluating treatment pathways and outcomes in real-world MM populations, including patients underrepresented in randomized controlled trials.

## Introduction

Multiple myeloma (MM) is a B-cell malignancy that disrupts bone marrow function, causing complications such as anemia, hypercalcemia, bone pain, fractures, and renal failure.^1–3^ Over the past two decades, treatment options have evolved significantly, with the emergence of novel therapeutic agents, as well as autologous stem cell transplants and better supportive care. Advances in therapy have extended survival, leading to a growing prevalence despite stable incidence.^4^ Therapeutic efficacy is primarily evaluated in randomized clinical trials (RCTs), which typically involve highly selected populations, underrepresenting elderly patients and those with comorbidities such as renal failure.^2,5^ Consequently, RCT findings may not fully reflect real-world effectiveness, highlighting the need for real-world data from broader patient populations.^6^

Large observational cohorts have been developed to address this gap.^6–8^ In France, the Epidemiology of Multiple MYeloma (EmmY) study is a non-interventional, longitudinal cohort study initiated in 2017 within the French-speaking *Intergroupe Francophone du Myélome* (IFM).^8^ Conducted in 75 specialist centers, EmmY monitors treatment practices and outcomes in MM. Recent analyses have documented shifts in therapy, including increased use of lenalidomide maintenance between 2017 and 2020^9^ and evolution in advanced line therapy.^10^ Although this observational cohort was not designed to be representative of all MM cases in a given area (mainly because participation of centers in this clinical network was voluntary and there was no sampling process for centers or patients), any differences in patient and center characteristics are likely to be negligible. The comparability of EmmY relative to the broader IFM network and the national MM population across France has, however, not yet been studied. The aim of this study was to compare patient, center and hospitalization (treatment) characteristics of newly diagnosed MM patients from participating centers of the EmmY cohort with characteristics of MM patients hospitalized in the IFM network and those hospitalized across the whole of France over the period 2017-2023.

## Materials and Methods

### Study Design and population

We analyzed national healthcare claims data from the French National Hospital Discharge database (*Programme de Médicalisation des Systèmes d’Information*, PMSI) to identify all patients hospitalized with a primary or related diagnosis of MM (ICD-10 code C90) across France.

Patients hospitalized for MM between 1 August 2017 and 31 December 2023 in a center participating in the EmmY cohort study were compared to two cohorts: patients hospitalized for MM in one of the 140 participating IFM centers (which included all EmmY centers) and patients hospitalized for MM in any center in France (including all EmmY and IFM centers). EmmY and IFM centers that did not participate in the networks for the full year and centers that had fewer than one patient hospitalized for MM per year were excluded from the analysis.

This study received a favorable opinion from the French Scientific and Ethical Committee for Health Research, Studies and Evaluations (CESREES) on 20/06/2024, No. 18096538 and from the French Data Privacy Authority CNIL HGT/MFI/AR2415177.

### Inclusion criteria for the Epidemiology of Multiple MYeloma (EmmY) cohort

Seventy-three IFM centers across France participate in EmmY (34 general hospitals, 26 university hospitals, 11 private hospitals/clinics, 1 military hospital, and 1 comprehensive cancer center) with ongoing patient recruitment at each center. Eligible patients are enrolled according to the following criteria: Adults aged ≥18 years, diagnosed with symptomatic MM and requiring systemic treatment (irrespective of the treatment line), initiating a new line of treatment during the annual predefined period from 1 October to 31 December each year.

### Data analyzed

We extracted from the PMSI database: center characteristics for all centers included in the study (EmmY, IFM, and all MM centers); hospitalization characteristics for all hospitalizations (any duration) with a primary or related diagnosis of MM (ICD-10 C90); and patient characteristics for all patients with at least one hospitalization during the study inclusion period in one of the included centers, with MM as the primary or related diagnosis.

### Statistical methods

Categorical variables were described by the frequency and percentage, and continuous variables were described by the mean, standard deviation, median, 1st and 3rd quartiles, minimum and maximum.

Standardized differences (SD) were used to quantify the magnitude of differences between groups as a relevant and interpretable measure independent of sample size. According to Austin,^11^ an imbalance can be considered when the absolute standardized difference exceeds S=1.96*square root (total N/N1*N2). However, in practice, and in line with the update by Schulte and Mascha,^12^ standardized difference values below 0.1 were considered negligible, even if smaller than the threshold proposed by Austin.

Standardized differences between the EmmY, IFM, and MM groups for patient, hospitalization, and center characteristics were estimated for the following characteristics:

- center type (private / public), location, and capacity;
- patient age, gender, and principal comorbidities; and
- hospitalization mode of entry and exit, duration, treatment received including chemotherapy, radiotherapy and stem cell transplants.

## Results

### Population

**Figure 1.**
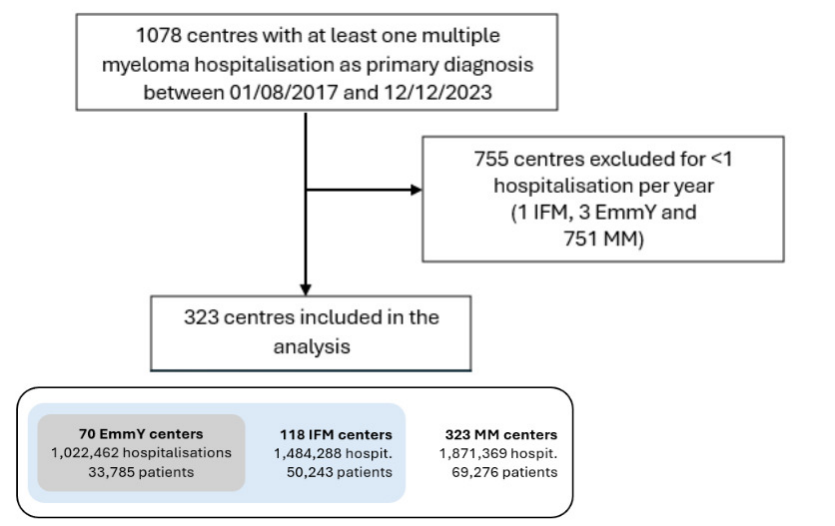
Flowchart of study inclusions for centers, hospitalizations and patients included in the EmmY,^13^ IFM,^7^ or all multiple myeloma^11^ (MM) cohorts over 2017-2023. ^13^EmmY - Epidemiology of Multiple Myeloma,^7^IFM – Intergroupe Francophone du Myélome,^11^MM – all centers in France who hospitalized at least one multiple myeloma patient per year during study period.

We identified 1,078 centers in France that could, in theory, have had at least one hospitalization for MM as the primary diagnosis during the study inclusion period (**Figure 1**). We then excluded 755 centers that did not meet inclusion criteria (751 MM centers that did not meet the minimum requirement of at least one patient per year, and one IFM center and three EmmY centers because they were not part of the IFM or EmmY network for the full study period), leaving 323 (100%) centers in France, among which, 70 were EmmY (21.7%) and 118 were IFM (36.5%) centers.

There were 1,871,369 hospitalizations for MM overall, including 1,022,462 (54.6%) in EmmY centers and 1,484,288 (79.3%) in IFM centers. This corresponded to a total number of 69,276 patients hospitalized in MM centers, 33,785 (48.8%) in EmmY and 50,243 (72.5%) in IFM centers.

### Comparing center, hospitalization and patient characteristics between the three cohorts

**Figure 2.**
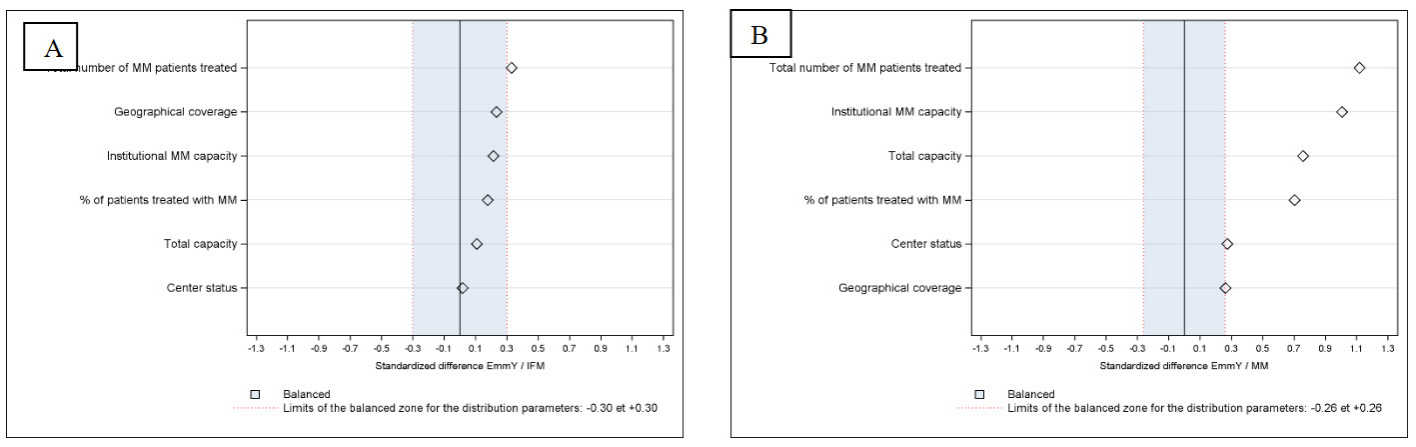
Standardized differences in center characteristics between EmmY and IFM (2A) and between EmmY and MM (2B) centers 2017-2023. ^13^EmmY - Epidemiology of Multiple Myeloma,^7^IFM – Intergroupe Francophone du Myélome,^11^MM – all centers in France who hospitalized at least one multiple myeloma patient per year during study period.

EmmY and IFM centers were larger than MM centers overall, with greater mean annual numbers of MM patients (207 for EmmY centers, 163 for IFM (SD 0.329) and 68 in MM centers) (SD 1.118) (**Table 1, Figure 2A and 2B**). The mean proportion of MM patients per center was higher in EmmY and IFM centers (2.72% and 2.35% (SD 0.178) respectively *vs.* 1.34% (SD 0.704) in MM centers), confirmed by SD greater than 0.1 (**Figures 2A and 2B**). EmmY centers treated more patients annually than MM centers (**Figure 2B**).

**Table 1.** Centre characteristics for patients hospitalized for multiple myeloma in France across EmmY^13^ centers, IFM^7^ centers, or all multiple myeloma centers^11^ (MM) over 2017-2023.

	*Centre*		
	*EmmY* *N=70 (21.7%)*	*IFM* *N=118 (36.5%)*	*MM* *N=323 (100%)*
Public center^14^	65 (92.9)	109 (92.4)	272 (84.2)
Private center	5 (7.1)	9 (7.6)	51 (15.8)
Mean total capacity (SD)	483,211 (237,507)	457,733 (236,065)	310,966 (216,517)
Mean annual capacity (SD)	75,328 (37,025)	71,356 (36,800)	48,476 (33,753)
Mean MM capacity (SD)	11,922 (7,826)	10,287 (7,403)	4,739 (6,352)
Annual MM capacity (SD)	1,858 (1,220)	1,604 (1,154)	739 (990)
Mean % of MM patients (SD)	2.72 (2.25)	2.35 (1.93)	1.34 (1.62)
Geographical region			
South East	16 (22.9)	26 (22.0)	74 (22.9)
West	17 (24.3)	25 (21.2)	66 (20.4)
Greater South West	10 (14.3)	22 (18.6)	67 (20.7)
Ile-de-France	10 (14.3)	21 (17.8)	49 (15.2)
East	8 (11.4)	12 (10.2)	35 (10.8)
North	8 (11.4)	11 (9.3)	20 (6.2)
Overseas departments & territories	ND^1^	ND^1^	12 (3.7)
Mean total MM patients (SD)	1,448 (980)	1,138 (902)	475 (744)
Mean annual MM patients (SD)	207 (140)	163 (129)	68 (106)

^13^ EmmY - Epidemiology of Multiple Myeloma.

^7^ IFM – Intergroupe Francophone du Myélome.

^11^MM – all centers in France who hospitalized at least one multiple myeloma patient per year during study period.

^1^ ND – not disclosed due to regulations for reporting on numbers <10 from the National Health Claims Database - SNDS.

^14^ Results are N (%) unless otherwise stated. SD – Standard Deviation

**Figure 3.**
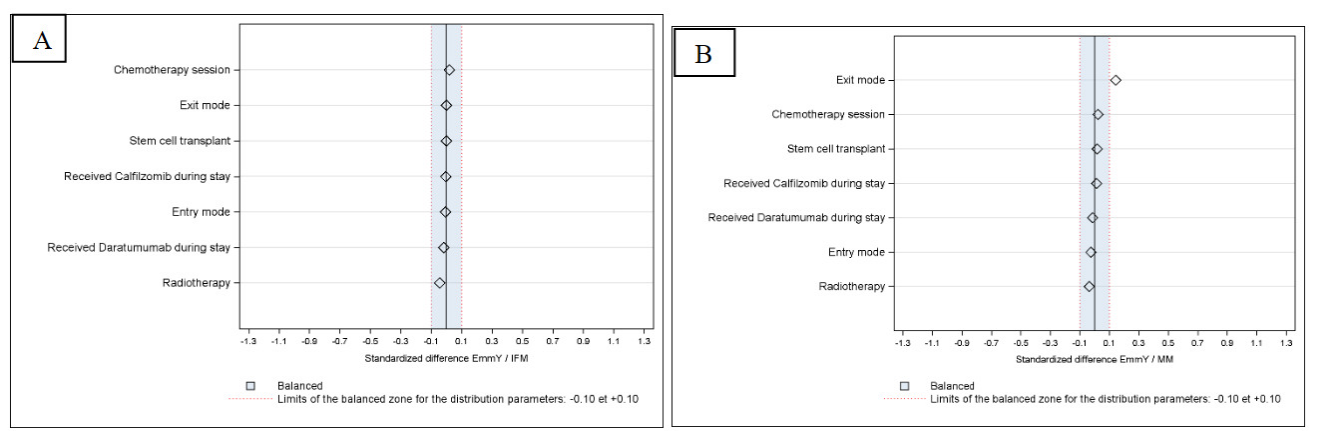
Standardized differences between hospitalizations in the EmmY,^13^ IFM^7^ (3A) or all multiple myeloma^11^ (MM) (3B) cohorts over 2017-2023. ^13^EmmY - Epidemiology of Multiple Myeloma,^7^IFM – Intergoupe Francophone du Myélome,^11^MM – all centers in France who hospitalized at least one multiple myeloma patient per year during study period.

There were no differences in hospitalization characteristics for hospitalizations in EmmY and IFM centers. The only significant SD greater than 0.1 at the hospitalization level was for exit mode, with fewer patients hospitalized in MM centers returning home after hospitalization and more being transferred during hospitalization (EmmY *vs*. MM SD 0.1421; EmmY *vs.* IFM SD <0.1) (**Table 2, Figure 3B**). As for treatment administration during hospitalization, there was no difference in the proportions of patients receiving chemotherapy, radiotherapy, carfilzomib and daratumumab or stem cell transplants across centers (SD<0.1)(**Figures 3A and 3B**).

**Table 2.** Hospitalization characteristics for patients hospitalized for multiple myeloma in France across EmmY^13^ centers, IFM^7^ centers, or all multiple myeloma^11^ (MM) centers over 2017-2023.

	*Center*		
	*EmmY* *N=1,022,462 (54.6%)*	*IFM* *N=*1,484,288 *(*79.3*%)*	*MM* *N=*1,871,369 *(100%)*
Entry mode:			
From home^1^	1,0151,78 (99.3)	1,472,996 (99.2)	1,853,749 (99.1)
Transfer	7,284 (0.7)	11,292 (0.8)	17,620 (0.9)
Discharge mode:			
Home	1,009,402 (98.7)	1,463,763 (98.6)	1,838,968 (98.3)
Transfer	9,723 (1.0)	15,317 (1.0)	24,272 (1.3)
Deceased	3,336 (0.3)	5,207 (0.4)	8,128 (0.4)
Chemotherapy	828,109 (81.0)	1,191,464 (80.3)	1,499,225 (80.1)
Radiotherapy	5456 (0.5)	13,671 (0.9)	15,589 (0.8)
Stem cell transplant	7,774 (0.8)	11,478 (0.8)	11,580 (0.6)
Carfilzomib administration	101,539 (9.9)	149,206 (10.1)	179,028 (9.6)
Daratumumab administration	238,752 (23.4)	357,773 (24.1)	447,762 (23.9)

^13^ EmmY - Epidemiology of Multiple Myeloma.

^7^ IFM – Intergroupe Francophone du Myélome.

^11^ MM – all centers in France who hospitalized at least one multiple myeloma patient per year during study period.

^1^ Data are N (%)

**Figure 4.**
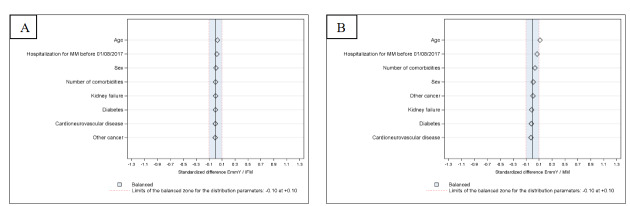
Standardized differences between patients hospitalized in the EmmY,^13^ IFM^7^ (4A) or all multiple myeloma^11^ (MM) (4B) cohorts over 2017-2023. ^13^EmmY - Epidemiology of Multiple Myeloma,^7^IFM – Intergoupe Francophone du Myélome,^11^MM – all centers in France who hospitalized at least one multiple myeloma patient per year during study period.

Patients hospitalized in EmmY centers were of similar median age to those hospitalized in IFM centers (SD<0.1) and younger than those hospitalized in MM centers (SD 0.114). No gender differences were observed. Proportions of patients with comorbidities were similar across cohorts (**Table 3**) (**Figures 4A and 4B**).

**Table 3.** Patient characteristics for patients hospitalized for multiple myeloma in France across EmmY^13^ centers, IFM^7^ centers, or all multiple myeloma^11^ (MM) centers over 2017-2023.

	*Center*		
	*EmmY* *N=33,785 (48.8%)*	*IFM* *N = 50,243 (72.5%)*	*MM* *N=69,276 (100%)*
Median age, years Age in categories	69	69	71
<65 years^1^	11,332 (33.5)	16,772 (33.4)	21,058 (30.4)
65-80 years	16,169 (47.9)	23,960 (47.7)	33,105 (47.8)
80 and over	6,284 (18.6)	9,511 (18.9)	15,113 (21.8)
Gender			
Male	18,371 (54.4)	27,173 (54.1)	37,324 (53.9)
Female	15,414 (45.6)	23,070 (45.9)	31,952 (46.1)
Hospitalization prior to 01/08/2017	6,532 (19.3)	9,327 (18.6)	11,539 (16.7)
Number of comorbidities			
0	21,939 (64.9)	32,471 (64.6)	44,549 (64.3)
1	7,862 (23.3)	11,776 (23.4)	16,011 (23.1)
2	2,788 (8.3)	4,176 (8.3)	5,998 (8.7)
3	879 (2.6)	1,343 (2.7)	2,008 (2.9)
4	260 (0.8)	389 (0.8)	581 (0.8)
5 or more	57 (0.2)	88 (0.2)	129 (0.2)
Other cancer	5,494 (16.3)	8,306 (16.5)	11,117 (16.0)
Diabetes	2,690 (8.0)	4,048 (8.1)	5,878 (8.5)
Renal failure	4,291 (12.7)	6,398 (12.7)	9,153 (13.2)
Cardio-neurovascular disease	4,072 (12.1)	6,120 (12.2)	8,922 (12.9)

^13^ EmmY - Epidemiology of Multiple Myeloma.

^7^ IFM – Intergroupe Francophone du Myélome.

^11^MM – all centers in France who hospitalized at least one multiple myeloma patient per year during study period.

^1^ Data are N (%)

## Discussion

To be representative, a real-world study conducted at the national level would require random sampling of both centers and patients, an approach that is rarely feasible in clinical research. Although this cohort was not designed to be representative, only a few differences were observed in center and patient characteristics between EmmY centers and all MM centers in France.

The main differences were the larger size of EmmY centers and the higher proportion of MM patients in these centers (>2%) compared to general MM centers (1%). This was expected as we established a very broad definition of centers treating MM in France, with the minimum study requirement for only one patient per year, resulting in the inclusion of some small centers in the overall MM cohort.

At the hospitalization level, with respect to modes of entry and discharge, and the treatments administered during hospitalization, there were no differences between hospitalizations in EmmY centers compared to in IFM centers. Only one small difference was observed between EmmY and MM centers with slightly more patients treated at the latter being discharged via transfer. This finding is easily explained: patients treated in generalized centers are more likely to be transferred to more specialized centers as required. However, it should be noted that while the SD is significant, the percentage difference is very small at 0.3%.

Importantly, at the patient level, we observed no marked SDs between patients treated at the three centers. Comparing our patient characteristics with those previously reported for the EmmY cohort from 2017-2020, we note that our patients are approximately similar in age, with 33.5% aged under 65 years in our cohort compared to 33.1% previously reported.^9^ Gender distribution is similar. We observed a lower rate of comorbidity-free patients in our study, at only 64.9%, compared to 71.6% in the study by Vincent *et al*. In addition, 31.6% of patients treated at EmmY centers in our study had 1-2 comorbidities compared to 22.2% in Vincent *et* al’s study. This may represent a rising prevalence of comorbidities across the population over time.

Demographic analyses from other cohorts have demonstrated similar findings. In the MYLORD^4^ study in France that used the national health claims database (SNDS) to identify MM patients, a slightly higher proportion (49.7%) of women was reported, with a median age of 71 years. In the interim analyses^14^ of the INSIGHT MM trial^6^ that included patients up to the 2017 cut-off from across 13 countries, only 15.9% of them were 75 years or older. One of the primary findings on that INSIGHT trial and in a recent systematic literature review of MM in developing countries^13^ was the heterogeneity in treatment patterns between countries and regions, making generalizations and comparisons more difficult. This same issue is not present in the EmmY cohort.

The importance of real-world evidence for advancing MM treatment is highlighted by our study, showing that over 20% of people hospitalized for MM were aged over 80 years, and they would not typically have been included in a treatment RCT if standard age-based patient exclusion criteria were applied. This is similar to a previous analysis of EmmY cohort patients, describing these patients as ‘older and frailer’ than those in clinical trials.^10^ The CONNECT-MM registry study has shown that 40% of patients were ineligible for RCTs based on common exclusion criteria, i.e., patients who had significantly lower survival rates.^15^ Furthermore, patients treated with novel agents in a large cohort study had a higher cytogenetic risk profile and more advanced disease stage compared to RCT populations.^16^ Consequently, they had a much lower response rate to treatment (53.3% in first-line and 25% in second-line) than reported by clinical trials.^16^

To generate meaningful evidence on treatment outcomes, studies must include populations that accurately represent the full spectrum of patients with MM, acknowledging that the characteristics of this population are changing continually as therapies advance and people with MM live longer with the disease. For example, in a study of hospitalizations for MM chemotherapy administration in the United States,^17^ a four-fold reduction in hospitalization rates was observed over 2002-2017. One of the strengths of the EmmY cohort is the standardized inclusion criteria and the homogeneity of therapeutic pathways in France, with all patients having access to the same therapies. Cohorts such as EmmY enable ongoing monitoring and documentation of these changes within a validated real-world context.

Our study has limitations. We only present data from patients with MM who are hospitalized. In a previous French study,^4^ when an algorithm was applied to identify patients from outpatient and community consumption healthcare data, compared to hospitalization-only data, the number of patients identified increased by 25%. Another limitation is that a one-off comparison may not capture changes over the year, although it is not very probable. In addition, the analysis compared only patients treated at EmmY centers, compared to IFM centers and those treated at centers in France overall. An additional analysis comparing patients included in the ongoing EmmY cohort^8^ with those of EmmY centers may be interesting.

## Conclusions

Results from large observational studies and registries such as EmmY are critical to generate robust real-world data to complement clinical trial findings and continue to improve outcomes for patients with MM. The present results describe the strength, homogeneity and comparability of the EmmY cohort, and demonstrate the utility and relevance of this cohort for future studies of MM in France.

### Authors’ Contribution

Conceptualization: OD, RG, AP, MM, FD, CL, AB, CH

Formal Analysis and Investigation: RG, FD, AB, LG

Methodology: OD, RG, AP, AB, CH

Supervision: OD, RG, AP, MM, BR, KBM, LV, TC, LF, CL, AB, CH

Writing – original draft: RG, PMS, AB

Writing – review & editing: All authors

### Competing Interests – COPE (Committee on Publication Ethics)

The following competing interests were declared:

Olivier Decaux: Medical writing funded by Sanofi, Amgen, BMS; Honoraria from Janssen, Celgene/BMS, Sanofi/ Takeda, GSK, Menarini-Stemline, and Pfizer; Consultancy fees for Advisory/Data Safety Monitoring Board for Janssen; Travel/meeting support from Roche, AbbVie, Janssen, and Celgene/BMS.

Aurore Perrot: Received honoraria from AbbVie, Amgen, BMS, GSK, Janssen, Pfizer, Sanofi, and Takeda.

Margaret Macro: Advisory board for Celgene/BMS, Janssen, Takeda, Amgen, GSK, Sanofi; Research funding from Janssen and Takeda; travel expenses covered by Janssen, Celgene/BMS, Takeda, Amgen, Sanofi.

Karim Belhadj Merzoug: Research grants from BMS; consultancy fees for advisory boards from BMS, Amgen, Janssen, and Leo Pharma; Speaker bureau for Amgen, Janssen, and Sanofi; travel expenses received from Abbvie, Sanofi, Takeda, and Janssen.

Laure Vincent: Expert boards, bibliographic presentations or writing educational documents for Janssen, BMS, Takeda, Sanofi; funding for conference expenses from Janssen, BMS, Sanofi, Pfizer, Takeda.

Thomas Chalopin: Received honoraria and served as a consultant for Bristol Myers Squib (BMS), GlaxoSmithKline (GSK), Janssen, Pfizer, Sanofi, Menarini-Stemline, and Takeda.

Laurent Frenzel: Is a consultant for Pfizer, Roche, CSL Berhing, SOBI, Biomarin

Arthur Bobin: Honoraria from Johnson & Johnson, Sanofi, Amgen, Pfizer, and Stemline Menarini.

Mohamed Mohty: Received honoraria from Janssen, Sanofi, BMS, Amgen, Takeda, GSK, Pfizer, Novartis, Jazz Pharmaceuticals, Menarini-Stemline, and Astellas.

Cyrille Touzeau: Served on advisory boards for and received honoraria from BMS, Amgen, Celgene, Janssen, Sanofi.

Murielle Roussel: Is a board member for GSK, Janssen, and Pfizer.

Cyrille Hulin: Honoraria from Amgen, Janssen, BMS, Pfizer, and AbbVie.

No competing interests were disclosed for all other authors.

### Ethical Conduct Approval – Helsinki

This study received a favorable opinion from the French Scientific and Ethical Committee for Health Research, Studies and Evaluations (*Comité éthique et scientifique pour les recherches, les études et les évaluations dans le domaine de la santé -* CESREES) on 20/06/2024, No. 18096538 and from the French Data Privacy Authority (*Commission nationale de l’informatique et des libertés -* CNIL) HGT/MFI/AR2415177 and was performed in accordance with the ethical standards as laid down in the 1964 Declaration of Helsinki and its later amendments.

### Informed Consent Statement

All authors and institutions have confirmed this manuscript suitable for publication.

## Data Availability

PMSI data are available upon request from the appropriate authorities.
